# Magnetic Resonance Imaging Characteristics of Ovarian Clear Cell Carcinoma

**DOI:** 10.1371/journal.pone.0132406

**Published:** 2015-07-10

**Authors:** Wei Wang, Jianhui Ding, Xiaoli Zhu, Yuan Li, Yajia Gu, Weijun Peng

**Affiliations:** 1 Department of Radiology, Fudan University Shanghai Cancer Center, Shanghai 200032, China; 2 Department of Oncology, Shanghai Medical College, Fudan University, Shanghai 200032, China; 3 Department of Pathology, Fudan University Shanghai Cancer Center, Shanghai 200032, China; University of Washington School of Medicine, UNITED STATES

## Abstract

**Purpose:**

To probe the magnetic resonance imaging (MRI) features of ovarian clear cell carcinoma (OCCC).

**Methods:**

This study retrospectively collected MRI data for 21 pathology-confirmed OCCCs from 19 female patients. The MRI findings were analyzed to determine the tumor size, shape/edge, shape and number of protrusions within the cyst, cystic or necrotic components, signal intensity (SI) and enhancement features.

**Results:**

The age of the 19 patients ranged from 28 to 63 years (mean age: 53 years). Unilateral tumors were found in 17 patients (17/19, 89%); the average size of all tumors was 10.8 cm. The tumors on MRI were classified into two categories: (a) “cystic adnexal mass with solid protrusions” in 12 (57%) and (b) “solid adnexal mass with cystic areas or necrosis” in 9 (43%). For group a, high to very high SI was observed for most tumors (10/12, 83%) on T1-weighted images (T1WIs), and very high SI was observed on T2-weighted images (T2WIs) for all 12 tumors. Most solid protrusions were irregular and few in number and exhibited heterogeneous intermediate SI on T1WIs and T2WIs and prolonged enhanced SI in the contrast study. All 9 OCCCs in group b were predominantly solid masses with unequally sized necrotic or cystic areas in which some cysts were located at the periphery of the tumor (4/9, 44%). The solid components in all 9 tumors showed iso- or slightly high SI on T1WIs, heterogeneous iso-high SI on T2WIs and heterogeneous prolonged enhancement. According to FIGO classification, 14 tumors (14/19, 74%) were stages I-II, and 5 (5/19, 26%) were stages III-IV.

**Conclusions:**

On MRI, OCCCs present as large unilateral multilocular or unilocular cystic masses with irregular intermediate SI solid protrusions or predominantly solid masses with cysts or necrosis at an early FIGO stage.

## Introduction

Ovarian cancer, one of the most lethal cancers in women, is second only to endometrial carcinoma as the most common gynecological malignancy. Approximately 90% of ovarian cancers are epithelial in origin, and subtypes of these tumors include serous, mucinous, endometrioid, clear cell, and undifferentiated tumors[1, 2]. Ovarian clear cell adenocarcinoma (OCCC) is a subgroup of primary epithelial ovarian carcinoma (EOC). In 1973, the World Health Organization (WHO) recognized OCCCs as a distinct histological type of epithelial ovarian neoplasm and defined OCCCs as tumors with clear cells growing in solid, tubular or glandular patterns and with hobnail cells lining the cysts and tubules[3]. The WHO updated the definition of OCCC in 2003 to describe a neoplasm composed of clear cells growing in a solid, tubular or papillary pattern with hobnail cells lining the tubules and cysts[4].

Despite diagnosis at early stages, OCCCs are biologically aggressive neoplasms. The presenting symptoms include abdominal discomfort, pain, distention and gastrointestinal symptoms. As OCCCs are remarkably platinum resistant[5], managing these tumors requires completely different surgical techniques and chemotherapy modalities[6]. The characterization of an ovarian mass is of the utmost importance in the preoperative evaluation of an ovarian neoplasm because it enables the surgeon to anticipate an ovarian carcinoma or predict the type of ovarian carcinoma prior to surgery so that adequate procedures can be planned[7].

Imaging plays a vital role in predicting treatment response and postoperative outcome and in monitoring recurrence[8], and magnetic resonance imaging (MRI) is effective for the diagnosis and accurate characterization of a wide spectrum of ovarian masses[8]. Indeed, MRI provides excellent tissue characterization and has demonstrated superiority to Doppler ultrasonography (US) and contrast-enhanced computed tomography (CT) for the accurate characterization of adnexal masses[9]. OCCCs typically manifest as a large, cystic, unilateral adnexal mass with one or more solid nodules protruding into the cyst lumen, similar to type I ovarian cancers[10]. However, the macroscopic characteristics of OCCC are not well defined by radiological examination because of the small number of imaging reports.

This study retrospectively collected a relatively large cohort (21 OCCCs in 19 patients) and characterized their MRI features.

## Materials and Methods

### Ethics statement

This retrospective study was approved by the Institutional Review Board of The Fudan Cancer Hospital. All patients provided written informed consent to participate in this study.

### Patients

From January 2010 to June 2014, 36 female patients with OCCCs were diagnosed by pathology at our institution; 19 of these patients, with 21 OCCCs, who were examined with MR imaging as a routine staging procedure before surgical treatment were recruited. All tumors were confirmed based on surgical excision of the lesion. None of the 19 patients had received prior treatment with chemotherapy, surgery or radiation. The medical records of the patients were reviewed to evaluate their clinical and general characteristics (Table 1). The most commonly used staging system for ovarian cancer is a surgically based system established by the International Federation of Gynecology and Obstetrics (FIGO)[11]. Based on these FIGO staging criteria, all of the ovarian neoplasms in this study presented as stages I-IV (Table 1).

**Table 1:** Clinical findings of 19 patients with OCCCs.

No./age	Symptoms and signs	Ascites	CA199(u/ml)	CA125(u/ml)	LN/Capsule/Vessel	Stage(FIGO)	Follow-up (months)
**1/54**	Pelvic lump incidentally detected on US	-	38.63	-	-/-/-	IIa	9
**2/50**	Pelvic lump detected on US	+500 ml malignant	-	-	-/+/-	Ic	28
**3/63**	Pelvic lump incidentally detected on US	-	-	111.10	-/-/-	Ic	2
**4/56**	Stomachache discontinuity	-	-	37.30	-/-/-	Ia	18
**5/47**	Pelvic lump for 5 years	+benign	100.40	69.35	-/+/-	Ic	8
**6/44**	Right lower quadrant vague pain, abdominal lump	+600 mlbenign	39.92	262.90	-/+/-	IIc	24
**7/59**	Frequent and urgent micturition, pelvic lump	++1000 mlmalignant	120.90	232.80	-/+/+	Ic	26
**8/56**	Abdominal distension for 2 months	++2000 mlmalignant	>1000	891.30	-/+/-	IIc	4
**9/59**	Pelvic lump detected on US	-	-	48.68	-/-/-	Ia	10
**10/47**	Abdominal distension for 2 months	+++4200 mlmalignant	193.10	1079	+/+/-	IV	4
**11/28**	Right lower quadrant pain	-	40.33	487.20	+/-/-	IIIc	6
**12/57**	Colporrhagia for 5 days	-	-	774.70	-/+/-	Ic	15
**13/61**	Thamuria, pelvic lump detected on US	-	627.4	528.4	-/-/-	Ic	27
**14/53**	Stomachache, pelvic lump	++1000ml malignant	27.81	361.50	+/+/-	IIIb	10
**15/56**	Pelvic lump incidentally detected on US	-	>1000.00	272.00	-/-/-	Ia	24
**16/57**	Pelvic lump incidentally detected on US	-	-	49.18	-/-/-	Ia	29
**17/51**	Pelvic lump, hypodynamia for 1 year	-	-	76.54	+/-/-	IIIc	1
**18/50**	Pelvic lump detected on US	-	47.40	102.60	+/+/-	IIIc	2
**19/56**	Pelvic lump detected on US	+100 mlbenign	-	92.20	-/-/-	Ia	9

Note: US: ultrasound; LN: Para-aortic lymph node; Ascites: from small amounts (+) to more (++) and in bulk (+++); normal range:-; Capsule/Vessel: invasion (+) or non-invasion (-) of the capsule or vessel of the ovary

### MRI acquisition

Sixteen patients were examined with a 3.0 T whole-body MR system (Signa HDx, GE Medical Systems), and three patients were examined with a 1.5 T twin-speed superconducting MR system (GE Signa with EXCITE II) using a phased-array eight channel body coil at 1–2 weeks prior to surgery. The MRI sequences included the following: T1-weighted axial spoiled gradient-echo imaging (SPRG) sequences (TR/TE, 230/2.3 ms; slice thickness, 5–6 mm; gap spacing, 1–1.5 mm; field of view (FOV), 35–39 cm; matrix, 320 × 224); fat-saturated T2-weighted axial fast spin-echo (FSE) sequences (TR/TE, 3220/103 ms; slice thickness, 5–6 mm; gap spacing, 1.5–2.5 mm; FOV, 35–39 cm; number of acquisitions, 2; matrix, 320 × 160) obtained from the aortic bifurcation to the femoral neck; sagittal T2-weighted FSE sequences without fat suppression obtained from the abdominal aortic bifurcation to the bottom of the buttocks (TR/TE, 2360/105 ms; slice thickness, 5–6 mm; gap spacing, 1–1.5 mm; FOV, 26 cm; matrix, 320 × 224); and intravenous contrast-enhanced images. The 3 T MRI scanner axial and sagittal (or coronal) contrast T1-weighted images with fat suppression were acquired using liver acquisition with a volume acceleration (LAVA) sequence obtained immediately after a bolus infusion of 0.1 mmol kg^-1^ of gadolinium-diethylenetriamine pentaacetic acid (Gd-DTPA, Magnevist, Bayer Schering Pharma AG, Berlin, Germany) from the renal hila to the femoral neck (TR/TE, 3.4/1.6 ms; slice thickness, 3–6 mm; gap spacing, 0 mm; FOV, 35–39 cm; matrix, 256 × 128). The 1.5 T MRI scanner contrast-enhanced images were obtained using T1-weighted FSPGR sequences (TR/TE, 230/1.5–2.5 ms; slice thickness, 5–6 mm; gap spacing, 1–1.5 mm; FOV, 35–39 cm; matrix, 320 × 224) with fat suppression.

### Image interpretation

The MR characteristics of all OCCCs were retrospectively analyzed by two experienced gynecological radiologists with knowledge of only the histological diagnosis. The images were reviewed on a picture archiving and communication system (GE Aw4.3 workstation).

The evaluated parameters included the tumor location, size, shape/edge, shape and number of protrusions within the cyst, cystic or necrotic components, and signal intensity (SI) on T2WIs, T1WIs and MRI enhancement features. Additionally, other findings, including intraperitoneal ascites, lymphadenopathy, and serum CA 125 and CA 199 levels, were recorded. The readers recorded the size of all ovarian masses on the axial images. The presence or absence of a tumor boundary was observed and evaluated in the contrast-delayed phase as a clearly defined or poorly defined border. The enhancement pattern of the tumor parenchyma was classified as homogeneous or heterogeneous and hypo-intense, iso-intense or hyper-intense compared with the uterine myometrium in each enhancement phase. A metastatic lymph node was considered in patients in whom the short-axis diameter of the lymph node was greater than 1 cm[12, 13]. In this study, OCCCs were grouped into the following two groups according to the appearance on MRI: (a) “cystic adnexal mass with solid protrusions”, which exhibited oval or irregular masses with vegetations protruding into the lumen (papillary projections) and were either few or many in number; (b) “solid adnexal mass with cystic areas or necrosis”, which was a solid mass with cystic areas or patchy necrosis. We defined “many protrusions” as a number > 3 and “few” as ≤ 3. We defined “small papillary projections” as having a size < 10 mm and “large papillary projections” as ≥ 10 mm.

### Statistical analysis

Group differences in mean CA 199, CA 125 and age between group a and group b were tested using the two sample t test. The χ^2^ test (Chi square test) was used to compare group differences in FIGO stage between groups a and b. Fisher’s exact test was used to compare the distribution of MRI T1 (T1WI signal) characteristics of the cysts between the two groups. The data analysis was performed using SPSS software version 19.0 (SPSS Inc., Chicago, IL, USA). P < .05 was considered to indicate a significant difference.

## Results

### MRI findings

**Fig 1:**
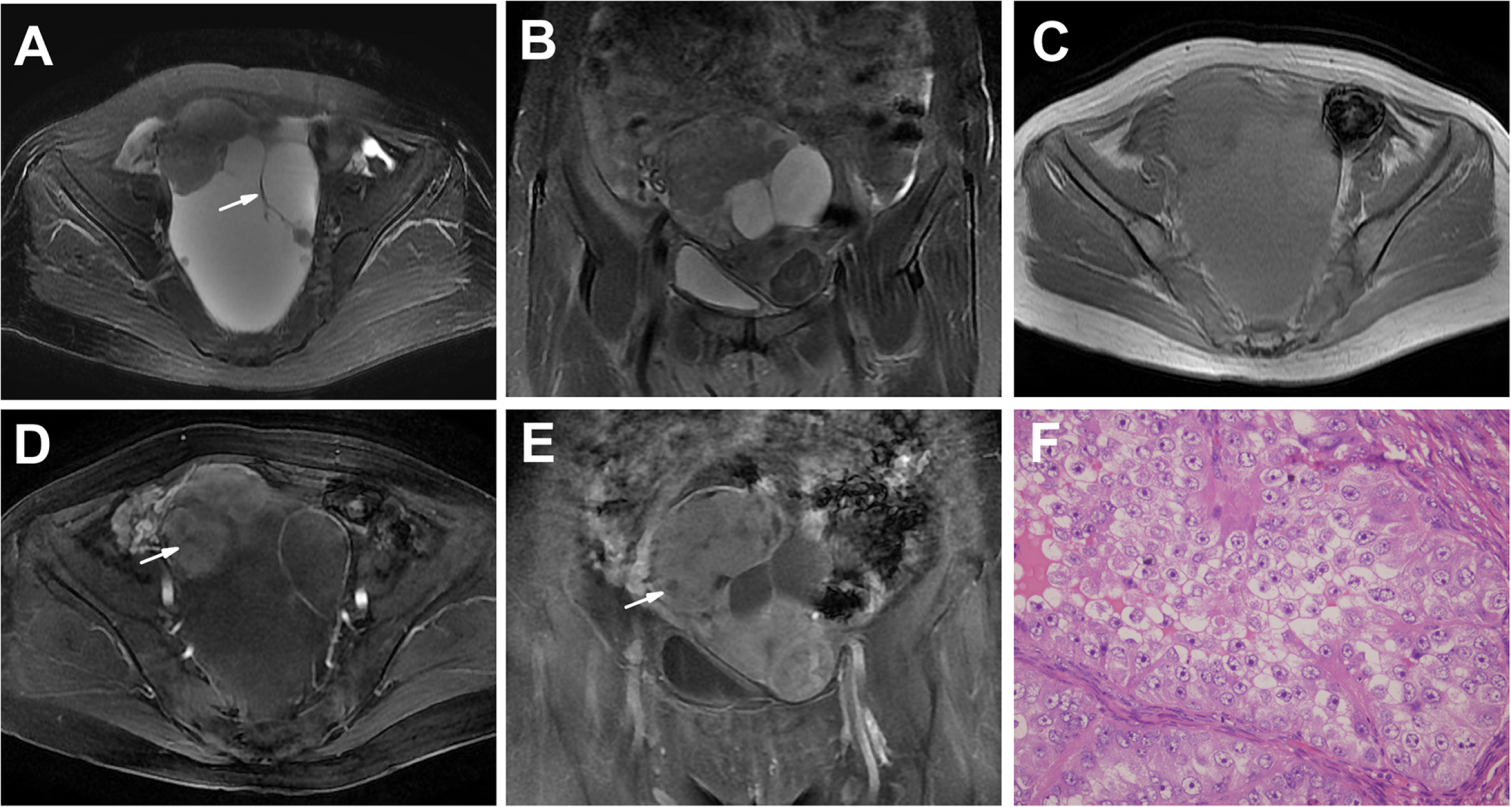
An OCCC in a 59-year-old woman (Patient 7 in Tables 1 and 2) with frequent and urgent micturition and a pelvic lump. **A-C** Axial (**A**), coronal T2WI (**B**) and plain (non-contrast) T1WI (**C**) showing a large and irregular unilateral, multilocular well-defined cystic mass with many irregularities and a few oval lumen solid protrusions. The septations were < 3 mm (arrow). The SI of the cyst was very high on T2WIs and high on T1WI. The solid protrusions had heterogeneous intermediate SI on T2WIs and T1WI (**A-C**). **D, E** Enhanced T1WIs showing markedly heterogeneous and prolonged enhancement solid protrusions, with nonenhanced portions (arrow) indicating effusion, as shown by pathology results. The thickened wall was enhanced. **F** The tumor shows a solid pattern with clear cells. (HE 40 & 10).

**Fig 2:**
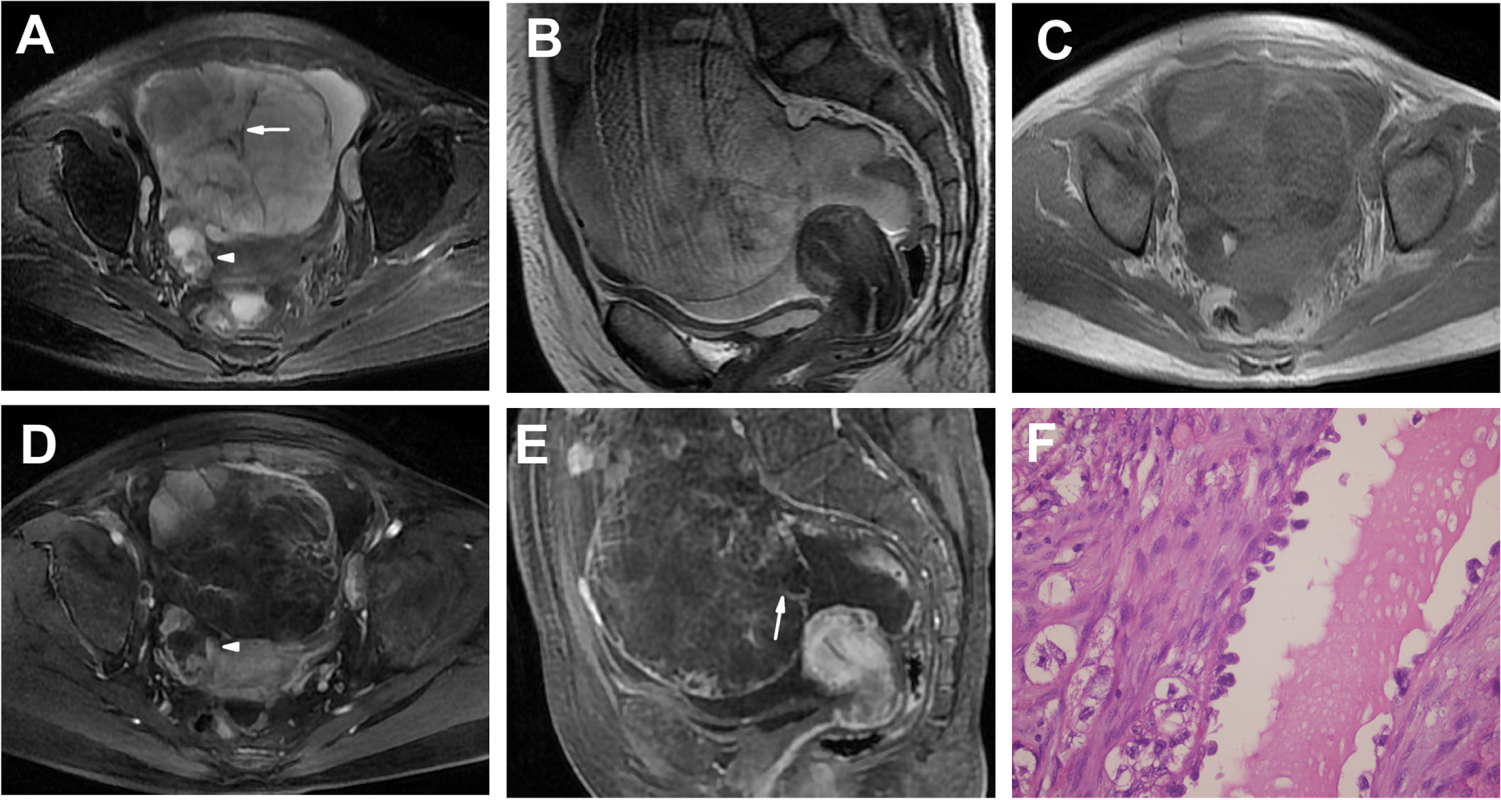
Bilateral OCCCs in a 47-year-old woman (Patient 10 in Tables 1 and 2) presenting with abdominal distension for 2 months. **A-C** Axial (**A**), sagittal T2WI (**B**) and plain (non-contrast) T1WI (**C**) showing a left-side, large, well-defined multilocular cystic mass with a few irregular lumen solid protrusions and many irregular septations (> 3 mm, arrow) and similar MRI findings of a right-side, small, multilocular cystic mass with protrusions (arrow head). The SI of the cyst was high on T2WIs and low on T1WI. The solid protrusions had heterogeneous iso-slightly high SI on T2WIs and iso-SI on T1WI. Bulk ascites were detected. **D, E** Enhanced T1WIs showing markedly heterogeneous and prolonged enhancement solid protrusions and septations (arrow). **F** The tumor shows a cyst lined with hobnail cells. Secretions were found in the lumen of the cyst. (HE 40 & 10).

**Fig 3:**
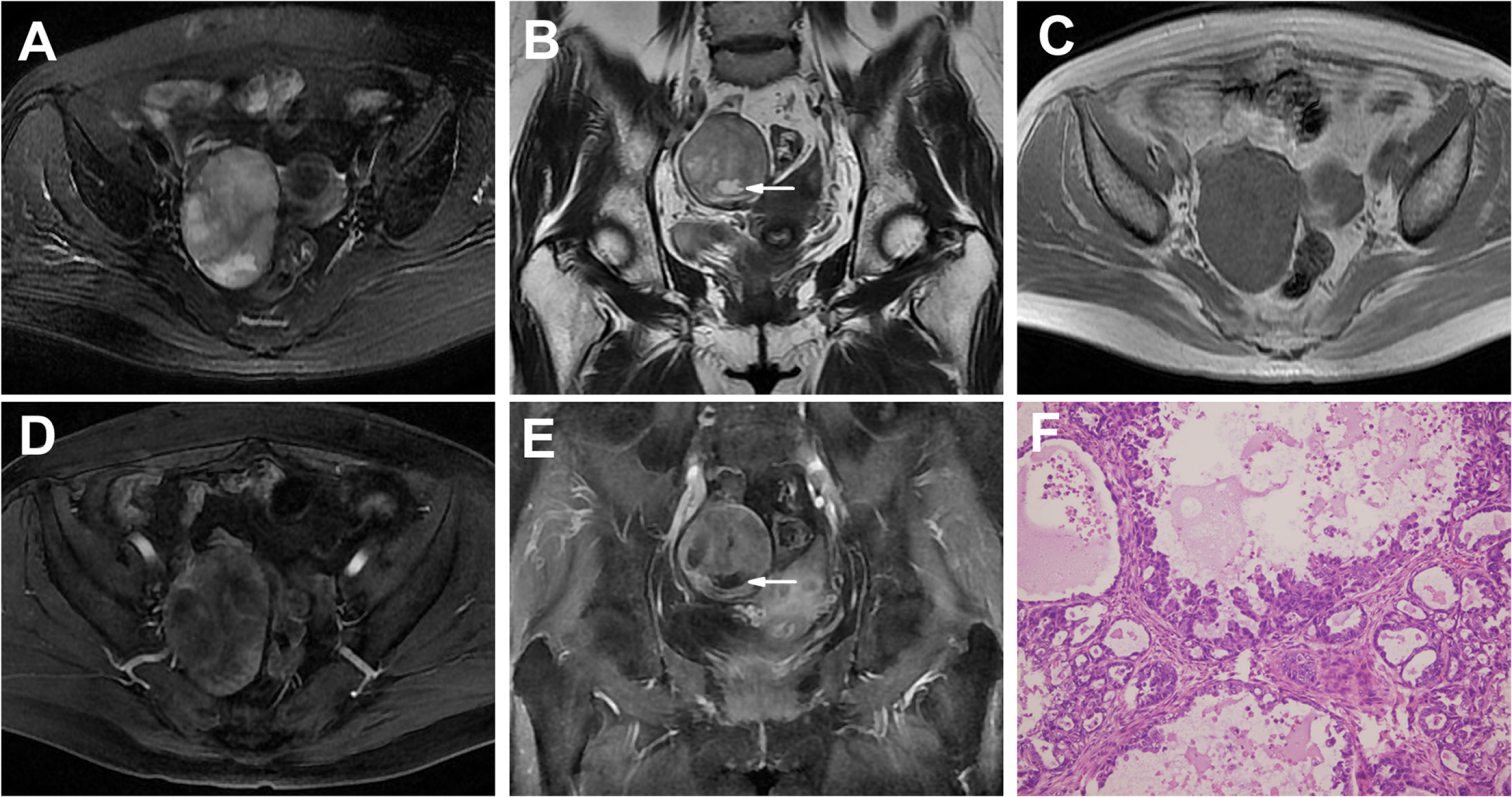
An OCCC in a 57-year-old woman (Patient 16 in Tables 1 and 2) with a pelvic lump incidentally detected on US. A-C Axial (**A**), coronal T2WI (**B**) and plain (non-contrast) T1WI (**C**) showing an oval well-defined solid mass with a few small cystic areas (high on T2WIs and low on T1WI, arrow) at the periphery segment of the tumor. The solid portion showed iso-SI on T1WI and high SI on T2WIs. **D, E** Enhanced T1WIs showing the markedly heterogeneous and prolonged enhancement solid mass with nonenhanced small cystic areas (arrow). **F** The tumor shows varying-sized cysts lined by papillary structure. The majority of tumor cells are oxyphilic cells with abundant eosinophilic cytoplasm. (HE 20 & 10).

**Fig 4:**
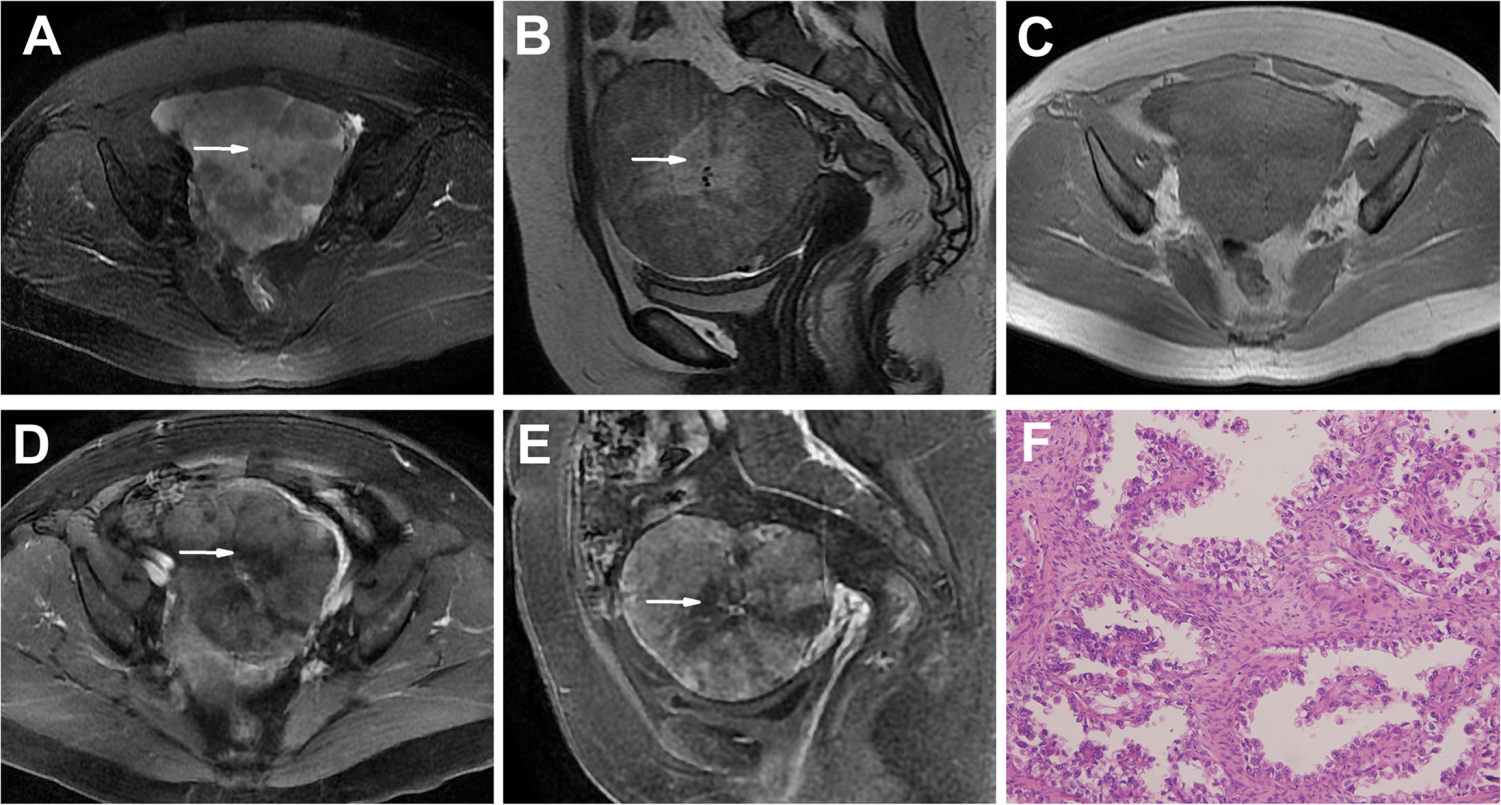
An OCCC in a 56-year-old woman (Patient 15 in Tables 1 and 2) with a pelvic lump incidentally detected on US. **A-C** Axial (**A**), sagittal T2WI (**B**) and plain (non-contrast) T1WI (**C**) showing a well-defined solid mass with central patchy, irregular necrosis areas (high on T2WIs and low on T1WI, arrow). The solid portion had intermediate SI on T2WIs and T1WI. **D, E** Enhanced T1WIs showing the heterogeneous and prolonged enhancement solid mass with central nonenhanced necrosis areas (arrow). **F** The tumor shows a papillary pattern with clear cells proliferating in a fibrous stoma. (HE 20 & 10).

The MRI characteristics of 21 OCCCs in 19 patients are summarized in Table 2, and representative patients are illustrated in Figs 1 to 4.

**Table 2:** MRI characteristics of 21 OCCCs in 19 patients.

No. /side	*Size (cm)	Shape/Edge	Cystic/solid	MR SI of the cyst		MR SI of the solid protrusion (first group)/solid portions (second group)			Pattern/Number of solid protrusion
				T1	T2	T1	T2	+C	
1/R	10×9	Oval/WD	Multi	High	Very high	Slightly high	Iso-	Enhanced	Irregular;inequality of size (+)
2/R	10.5×12	Oval/WD	Uni	Very high	Very high	Slightly low	Iso-	Slightly hetero-enhanced	Irregular; inequality of size (+)
3/L	7.5×7	Oval/WD	Multi	Low	Very high	Hetero-slightly high	Hetero-iso-high	Enhanced	Irregular; inequality of size(+)
4/L	10.5×8	Oval/WD	Uni	High	Very high	Slightly low	Hetero-slightly high	Enhanced	Irregular; inequality of size (++)
5/R	6×4.5	Irreg./WD	Multi	Very high	Very high	Slightly low	Iso-	Hetero- enhanced	Irregular; inequality of size (+)
6/L	15×11	Irreg./WD	Uni	High	Very high	Slightly high	Hetero-iso-slightly high	Enhanced	Irregular; inequality of size (+)
7/R	18×11	Irreg./WD	Multi	High	Very high	Iso-(high)	Iso-(high)	Enhanced	Irregular inequality of size (+ +)
8/R	21×16	Irreg./WD	Multi	Low	Very high	Hetero- iso-slightly high	Hetero-iso- high	Enhanced	Irregular; inequality of size (++)
9/L	10×7.5	Irreg./WD	Multi	Very high	Very high	Iso-	Hetero-iso-	Enhanced	Irregular; inequality of size (++)
10/L	13×11	Irreg./WD	Multi	Hetero-high low	High	Iso- slightly high	Iso-	Hetero- delayed enhanced	Irregular;inequality of size (+)
10/R	2.5×1.5	Irreg./WD	Multi	Iso- low very high	Iso-very high	Slightly high	Iso-	Hetero- delayed enhanced	Irregular;inequality of size (+)
11/R	10×8	Irreg./WD	Uni	Low	Very high	Slightly high	Hetero-slightly high	Hetero- delayed enhanced	Irregular;inequality of size (+)
12/L	9×5	Oval/WD	Solid with large cyst	Very high	Very high	Iso-	Slightly high	Hetero- slightly and delayed enhanced	
13/L	9.3×8.5	Irreg./ID	Solid with small cyst	Low	High	Iso- low	High	Hetero- delayed enhanced	
14/L	18×14	Irreg./WD	Solid with small cyst	Low	High	Slightly high	High	Hetero- delayed enhanced	
15/L	10×9	Irreg./WD	Solid with a few necrosis	Low	High	Iso-	Iso-	Hetero- delayed enhanced	
16/R	9×6	Oval/WD	solid with a few cyst	Low	High	Iso-	Hetero- high	Hetero- delayed enhanced	
17/L	10×8	Irreg./WD	Solid with many cystic or necrosis areas	Low	High	Hetero- Low and high	Hetero- high	Hetero- delayed enhanced	
17/R	5×5	Irreg./ID	Solid with cysts	Iso-	High	Iso-	Hetero- high	Hetero- delayed enhanced	
18/L	12×8	Irreg./WD	Solid with cyst	Low	High	Iso-	Hetero- High	Hetero- delayed enhanced	
19/R	10×6	Oval/WD	Solid with large necrosis	Low	Hetero- high	Iso-	High	Hetero- delayed enhanced	

Note:*Size: the largest tumor diameter observed on axial scans. Irreg., irregular; WD, well-defined; ID, ill-defined; Multi,multilocular cystic mass; Uni,unilocular cystic mass; Iso-,isointensity signal; High, high signal intensity; Low, low signal intensity; Very high, isointensity of fat on T1WIs; Very high on T2WIs, same or higher than the intensity of urine or spinal fluid; Inequality of size +, a few; Inequality of size + +, many in number; Signal intensity, SI; Hetero-, heterogeneous

The tumor presentation was unilateral in 17 patients (17/19, 89%) and bilateral in two patients (2/19, 11%; Patients 10 and 17). Nineteen OCCCs had smooth margins (19/21, 90%), whereas two tumors had obscure margins. Seven tumors were oval, and 14 (14/21, 67%) were irregular. Each of the 21 tumors was surrounded by a fibrous capsule or a false capsule.

#### “Cystic adnexal mass with solid protrusions” (12 tumors)

Twelve tumors (12/21, 57%) appeared as unilocular cystic masses (n = 5) or multilocular cystic masses (n = 7) with solid lumen protrusions (Figs 1 and 2). The number of solid protrusions in the cystic masses ranged from only a few (n = 8 tumors) to many (n = 4 tumors), and the size of the solid protrusions ranged from small to large. The solid protrusions were round and oval or irregular in one tumor (Fig 1A, Patient 7) and irregular in the other 11 tumors (Figs 1 and 2A–2E). The septations in the majority of the multilocular cystic masses were limited in number and < 3 mm in thickness (Fig 1A and 1D); an exception was found for those of the tumors in Patient 10 (Fig 2A and 2E), in which the septations were thickened and numerous. One large cystic area with a few small loculi was detected in 6 (6/7, 86%) multilocular cystic masses (Fig 1A and 1D). Partial thick-walled masses accounted for 83% of the masses (10/12) (Figs 1 and 2D and 2E).

On T1WIs, the cyst SI was low, high and very high in 3, 4 and 3 tumors, respectively (Figs 1 and 2C), whereas the cyst SI was very high in all 12 OCCCs on T2WIs (Figs 1 and 2A and 2B). In the two tumors in Patient 10, one T1WI revealed heterogeneous SI (hyperintense and hypointense) in the loculi (Fig 2C), presenting with a “honeycomb” appearance, and most of the cysts exhibited a high SI on T2WIs (Fig 2A and 2B), indicating different components within different loculi. There was an oval abnormal SI area (very high SI on T1WIs and low SI on T2WIs) in the right OCCC of Patient 10 that suggested a hemorrhage (Fig 2C).

For all 12 tumors, the solid protrusions had heterogeneous SI on T1WIs and T2WIs. On T1WIs, the SI of the protrusions was slightly low and iso- or slightly high in 3 and 9 tumors, respectively; on T2WIs, the SI of the protrusions was iso- or slightly high in all 12 tumors. In the contrast study, the protrusions showed marked heterogeneous enhancement in 11 masses (Figs 1 and 2D and 2E), slight heterogeneous enhancement in one tumor, and prolonged enhancement in 12 tumors. Unenhanced portions were detected in many protrusions, particularly those of large size, which were demonstrated by the pathology examination to be effusion or necrosis (Fig 1D and 1E).

#### “Solid adnexal mass with cystic areas or necrosis” (9 tumors)

Most of the masses in this group were irregular (6/9, 67%), though 3 tumors were oval (3/9, 33%). In this group, all 9 tumors were predominantly solid masses with unequally sized cystic areas or necrosis, with the cystic areas in the periphery region in 4 masses (4/9, 44%) (Fig 3A and 3E). A solid mass with a large cystic area was observed in 1 tumor. Patchy slit-shaped necrosis in the central tumor region was detected in 2 masses (Fig 4A–4E).

The solid portions of the 9 masses showed iso- or slightly high SI on T1WIs and heterogeneous iso-high SI on T2WIs; in the contrast study, the tumors displayed heterogeneous and delayed enhancement.

There were several high-SI areas on T1WIs and T2WIs, indicating hemorrhaging and patchy necrosis within the central region and the cystic areas at the periphery region of the tumor (Patient 19). In one patient (Patient 18), an endometrial cyst in the right ovary shown by pathology examination was found to present on MRI with high SI on T1WIs and T2WIs.

There was a significant difference in the MRI T1 signal characteristics of the cysts between the two groups (*P* < .05, Table 3).

**Table 3:** Statistical results of clinical and imaging features between cystic and solid tumors.

	Cystic tumor(n = 11)	Solid tumor (n = 8)	p
**age**	52.0±7.5	55.1±3.6	0.309
**CA199**	219.0±349.0	425.7±473.3	0.425
**CA125**	357.7±385.4	282.1±260.4	0.647
**T1s**			0.020
** Low**	3	7	
** High**	8	1	
**FIGO stage**			0.192
** I**	6	5	
** II**	3	0	
** III**	1	3	
** IV**	1	0	

**Note:** T1s, T1 signal

### Clinical characteristics according to the two groups

The age of the patients ranged from 28 to 63 years (mean age: 53 years). The majority of the OCCC patients had stage I/II disease (14/19, 74%), with most of them having stage I disease (11/19, 58%).

The histopathological features of the tumors from six representative patients are shown in Figs 1–4. The size of the tumors ranged from 2.5×1.5 cm to 21×16 cm, with a median size of 10.8 cm. The OCCCs display pure or mixed tubulocystic, papillary and solid patterns. The most common cell types included clear and hobnail cells and other cells, such as eosinophilic cells. There was no significant difference in FIGO stage between the two groups (*P* > .05, Table 3).

The CA199 serum level (normal, < 27 U/mL) was elevated in 11 patients, and the CA 125 serum level (normal, < 35 U/mL) was elevated in 17 patients. There was no significant difference in serum CA199, CA125 or age between the two groups (*P* > .05, Table 3). Four patients presented with uterine adenomyosis (Patient 3), pelvic endometriosis (Patients 12 and 19) or endometriosis of the right ovary (Patient 18).

## Discussion

Compared with other histological types, OCCC accounts for 5%-10% of all ovarian tumors[14] and has unique molecular characteristics as well as specific biological and clinical features[14, 15]. Although reports on the imaging features of OCCCs are limited, common MRI findings have shown that OCCCs are unilateral or bilateral, thick-walled, unilocular or multilocular cysts with peripheral mural soft-tissue nodules (frequently round and few in number)[10]. Similar appearances have been found by multidetector CT[16].

OCCCs frequently present predominantly as a large pelvic mass, and they rarely occur bilaterally. In our study, the majority of tumors were unilateral, and the size ranged from 2.5×1.5 cm to 21×16 cm, which was in agreement with reports indicating that the size of masses generally ranges from 3 to 20 cm[17, 18].

The tumors in the first group (group a) exhibited the typical imaging features of OCCCs[10, 14, 16]. One study indicated that a unilocular large cyst with solid protrusions is a common feature of OCCCs on MRI[10], and in another report, all OCCCs showed a predominantly cystic appearance with masses protruding into the lumen[16]. Most of the tumors (12/21, 57%) in our study had this type of presentation; however, multilocular masses (8/12, 67%) were found more frequently, which was inconsistent with other reports[10, 16]. In the present study, there were few septations (< 3 mm in thickness) in the multilocular cystic masses in the majority of tumors that presented with one large cyst and a few small loculi; a partial thick-wall was common. The margins of the 12 cystic tumors were well-defined and smooth, which was in agreement with a report[10].

In the 12 cystic tumors, the cysts showed very high SI on T2WIs, which was in accordance with a report[10]. However, different from the results of this report (30%)[10], the cysts in 8 tumors in our study had high to very high SI on T1WIs (67%). Most of the cysts exhibited high SI on T1WIs and T2WIs, which might be a result of the bulk of the mucosal fluid or hemorrhaging within the cysts.

There were a few solid protrusions within most of the cystic masses (8/12, 67%), and the majority of the solid protrusions were irregular, which was not consistent with a report that found that protrusions were frequently round[10]. The solid protrusions in our study were small or large in size, and solid components displayed heterogeneous intermediate SI. In fact, most of the solid protrusions had heterogeneous iso-high SI on T1WIs or T2WIs, and all of the protrusions had heterogeneous and prolonged enhancement on the contrast study, which differed slightly from a report in which no enhanced protrusions were detected[10]. In our study, some unenhanced areas were detected within partial protrusions, which on pathology results appeared to be effusion and necrosis; in contrast, one report suggested that the partial nonenhanced portion of solid protrusions might be myxoid changes[10].

Solid, nonfatty, nonfibrous tissue is the most powerful predictor of malignancy[19]. Thus, the tumors in the second group (group b) were consistent with malignancy. Most of the masses were irregular. The MRI findings in this group were slightly complicated. Only a small proportion of solid masses were detected previously (2/12, 17%)[10]; conversely, solid OCCCs accounted for 48% (10/21) of the tumors in our study. Therefore, this type of solid tumor deserves attention. All of the 9 OCCCs in group b were predominantly solid masses, and 4 of the masses (4/9, 44%) presented with small cystic areas or necrosis at the periphery of the tumor, similar to reported results[10]. On these predominantly solid masses, slit-shaped necrosis was detected in the central segment of the masses in 2 tumors (2/9, 22%). A large cystic area was also demonstrated in 1 tumor (1/9, 11%). To our knowledge, these two types of MRI features have not been reported to date. The solid components exhibited iso- or slightly high SI on T1WIs, heterogeneous iso-high SI on T2WIs in most tumors and heterogeneous prolonged enhancement in all 9 tumors in our study.

There was a statistically significant difference between the groups for T1WIs; the cysts in group a had high SI, but low SI was noted in group b. These results indicated that the contents of the cysts in the two groups were different: mucosal fluid or hemorrhaging within the cysts in group a and necrosis or cystic areas in group b. Despite the different cyst contents and distinct MRI characteristics between the cyst and solid groups, there were no significant differences in clinical features.

Many ovarian tumors manifest as large pelvic masses. Thus, differential diagnosis includes other EOC types, primary tumors, and metastatic disease.

Papillary projections are not specific features of OCCCs because they can be detected in serous or mucinous borderline ovarian tumors (BOTs) and carcinomas. Patients with borderline tumors are younger than those with OCCCs and are frequently diagnosed at an earlier stage[20, 21]. Mucinous BOTs (MBOTs) are more frequently multilocular, with loculi of different SI that have a “stained-glass appearance” as well as a high number of septa (>10), which are commonly described as irregular and thickened[22, 23]. Serous BOTs (SBOTs) are more frequently bilateral, with a higher amount of solid tissue, predominantly corresponding to vegetations with a high T2 signal[22]. In the present study, most of the OCCCs were unilateral masses in patients with stage I/II disease. The presence of a multilocular or unilocular high SI cystic unilateral adnexal mass with thick walls and few septations (< 3 mm) characterized the first group of OCCCs. Primary mucinous ovarian carcinomas are rare[8], and most appear as large, unilateral, multilocular cystic tumors with solid nodules and enhancing septa[24, 25]. Most mucinous carcinomas (80%) are stage I tumors, with an excellent prognosis after treatment[8]. However, in our study, these features were difficult to distinguish in multilocular cystic OCCCs. Pseudomyxoma peritonei might be observed in mucinous adenocarcinomas. The second group in our study included MRI features that were not typical of OCCCs, for which there are few related reports, making a differential diagnosis somewhat difficult.

Metastatic ovarian adenocarcinomas are most frequently in the stomach, appearing as bilateral lobulated and predominantly solid ovarian masses, with the ovaries retaining an oval shape[26, 27], and most primary tumors can be detected simultaneously. The enhancing patterns on dynamic contrast analysis indicated that metastatic tumors show a plateau after a rapid-increase pattern[26]. Peritoneal carcinomatosis may also be detected, and 2/3 of the patients presented with ascites. These features could be relatively simple to differentiate from the features of OCCCs.

This study had several limitations. First, because OCCC is an uncommon ovarian carcinoma subtype, a selection bias was inevitable. Second, because this study was a retrospective study without a carefully designed radiological plan, only routine MR images were available, and different MRI systems were used (3.0 T and 1.5 T). Thus, larger samples with advanced MRI techniques, including diffusion-weighted imaging, perfusion-weighted imaging and MR spectroscopy, are recommended to improve the imaging diagnostics of these tumors. Although this study provides important MRI findings, the imaging features of OCCCs remain complicated, and the final diagnosis should be based on a microscopic pathology examination.

In conclusion, OCCCs typically presented as a unilateral large cystic mass with a few irregular solid protrusions; however, a solid adnexal mass with cystic areas and necrosis were also found to be important imaging features of OCCCs. Thus, OCCCs should be considered in patients characterized by the following: (1) a 40- to 60-year old female patient with a unilateral large cystic and solid mass in an early stage; (2) two types of MRI features, a) multilocular or unilocular high SI thick-walled cystic adnexal mass with few septations (< 3 mm) presenting with intermediate SI and heterogeneous prolonged enhanced irregular solid protrusions and b) a heterogeneous prolonged enhanced predominantly solid adnexal mass with cystic areas or necrosis; (3) elevated serum levels of CA 125 and CA 199.
